# Bidirectional relationships between disability and cognitive decline: a 6-year longitudinal study

**DOI:** 10.1186/s12877-025-06511-6

**Published:** 2025-11-11

**Authors:** Tsung-Hsuan Hung, Tzu-Yun Wang, Hung-Chang Chou, Ching-Ju Chiu, Chia-Ning Lee, Huai-Hsuan Tseng, Kao Chin Chen, Yan-Jhu Su, Andrew Alberth, Yen Kuang Yang, Tsung-Yu Tsai

**Affiliations:** 1 Department of Medical Education, Kaohsiung Chang Gung Memorial Hospital, No.123, Dapi Rd, Niaosong District, Kaohsiung, 833 Taiwan; 2 Department of Psychiatry, National Cheng Kung University Hospital, College of Medicine, National Cheng Kung University, No.138, Shengli Rd, North District, Tainan City, 70428 Taiwan; 3 Department of Pharmacy and Master Program, Tajen University, No.20, Weixin Rd, Yanpu Township, Pingtung, 907 Taiwan; 4 Institute of Gerontology, College of Medicine, National Cheng Kung University, No.1, University Rd, North District, Tainan City, 701 Taiwan; 5 Department of Gerontology, University of Massachusetts Boston, 100 Morrissey Blvd, Boston, MA 02125 USA; 6 Institute of Behavioral Medicine, College of Medicine, National Cheng Kung University, No.138, Shengli Rd, North District, Tainan, 704028 Taiwan

**Keywords:** Disability, Cognitive change, Bidirectional relationships, Older adults

## Abstract

**Background:**

Disability and cognitive impairment are two significant age-related health issues in older adults. This study aims to simultaneously identify the bidirectional relationship between disability and cognitive impairment, and further pinpoint which one holds a more pivotal role in the relationship.

**Methods:**

A total of 628 participants aged 66.0 (SD = 7.3) were retrieved from the 2000 and 2006 Social Environment and Biomarkers of Aging Study (SEBAS). Cognitive function was assessed using the modified Short Portable Mental Status Questionnaire (SPMSQ). Disability was measured as a count of activity of daily living, instrumental activity of daily living, and mobility scales. Structural equation models with cross-lagged analysis were used to examine the temporal relationship between cognitive impairment and disability over six years.

**Results:**

The cross-lagged model was well constructed (χ2 (9) = 34.5941, *p* < 0.0001, RMSEA = 0.0673, CFI = 0.99, SRMR = 0.03). Baseline disability significantly predicted future cognitive impairment (β=−0.25, *p* < 0.05), and baseline cognition significantly predicted future disability (β=−0.03, *p* < 0.05). Fisher’s Z test indicated a stronger pathway from disability to future cognitive impairment compared to cognitive impairment to future disability (*p* < 0.001).

**Conclusion:**

This longitudinal study suggested that disability and cognition change were bidirectional, with disability showing a stronger impact on future cognitive impairment. Further dynamic investigations and mechanisms between cognitive change and disability are warranted.

## Background

 The World Health Organization (WHO) predicts that by 2030, approximately one in six people globally will be over the age of 60. The older population, both in terms of size and proportion, is increasing in nearly every country [1]. This demographic shift highlights the importance of addressing age-related health issues. One prevalent age-related health concern is disability, defined as an individual’s inability to engage in substantial and gainful activity due to medically physical or mental impairment [2]. On average, older adults in Taiwan experienced 5.53 years of moderate to severe disability before death [3]. It is estimated that disability affects approximately 16% of the world’s population, and the ratio of non-disabled older populations to disabled older populations is expected to decrease from 3.2 to 1 in 1985 to 2.5 to 1 in 2060. This trend presents an unavoidable challenge for the long-term healthcare system and the associated social support network [4, 5]. Another noteworthy, age-related health issue is cognitive impairment, characterized by difficulties in concentration, memory, and decision-making. The global incidence of mild cognitive impairment stands at around 15.56% among community-dwelling individuals 50 years and older worldwide [6]. People with cognitive impairment may struggle with remembering family members’ names, feel disoriented in familiar surroundings, gradually withdraw from social participation, and suffer adverse effects on both physical and mental health [7].

Disability and cognitive impairment are interrelated conditions that often co-occur in older adults, leading to a synergistic increase in risks of falls, injury, and mortality [3, 8]. Understanding the causal and reinforcing relationship between these two is essential for identifying intervention points that could slow their progression. It is intuitive to expect a connection, as both share underlying mechanisms, such as chronic inflammation and malnutrition [9–11]. However, longitudinal studies exploring this relationship remain inconclusive. Some suggest a bidirectional relationship [12–15], where each condition predicts the progression of the other, while others indicate a unidirectional effect, with one condition forecasting declines in the other [16, 17]. Additionally, there is significant variability in how disability is measured across studies. In this study, we consider disability as a multifaceted construct encompassing various dimensions of functions. To capture the full scope of disability, it will be assessed with a more comprehensive approach, rather than relying on a single parameter.

To clarify the associations between cognitive impairment and disability, the present study simultaneously examined cognition and disability by analyzing a 6-year longitudinal study conducted in Taiwan. There were two aims to identify the relationships between cognitive function and disability. First, we hypothesized that cognitive function and disability were bidirectional. Second, considering the broader impact of disability on health aspects, we further hypothesized that the influence of disability on future cognitive impairment was stronger than cognitive impairment on future disability. Understanding the directionality in associations between cognitive function and disability could guide future research and inform therapeutic interventions aimed at mitigating the progression of each condition.

## Methods

### Participants

**Fig. 1:**
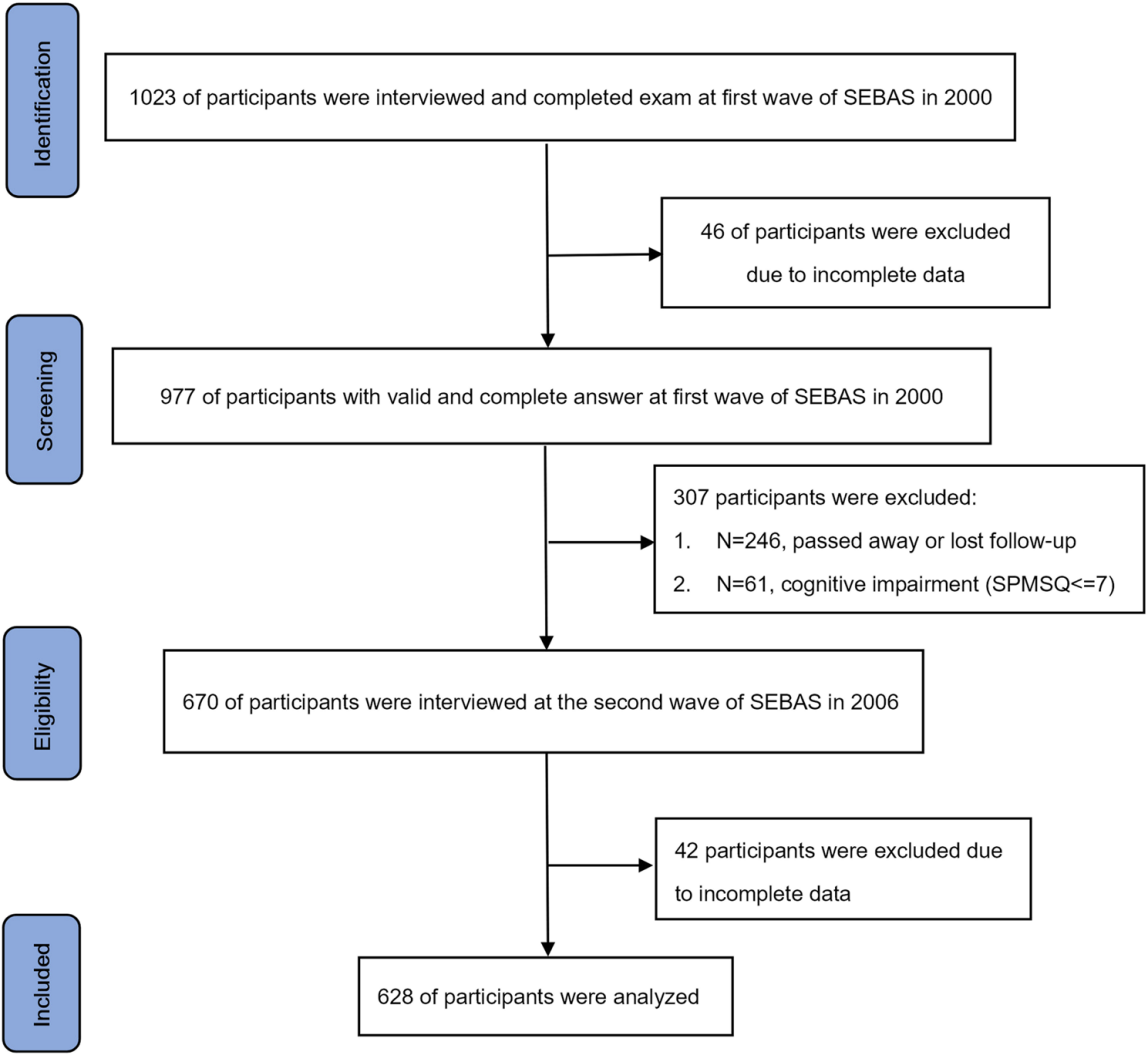
Flow chart of sample selection, participation, and attrition for SEBAS 2000 and 2006

This study included 689 participants from two waves of the Social Environment and Biomarkers of Aging Study (SEBAS), conducted in 2000 and 2006 [18]. SEBAS is an extension of the Taiwan Longitudinal Study on Aging (TLSA), which began in 1989 with periodic follow-ups every 3–4 years, involving a nationally representative sample of adults aged 60 and above. The first SEBAS wave in 2000, comprising 1023 participants, was derived from a subset of 1999 TLSA participants. For the present study, participants were eligible if they took part in both hospital-based health examinations and interviews during the 2000 and 2006 study waves and had data available for each variable of interest. A total of 977 participants met these criteria. To ensure the reliability of the self-report questionnaires, 61 participants were excluded due to potential cognitive function impairments at baseline, indicated by a score of 7 or lower on the Short Portable Mental State Questionnaire (SPMSQ). The second SEBAS wave in 2006 followed a similar protocol, collecting data on health status, health behavior, exposure to stressors, and social relationships. After accounting or 246 participant deaths or loss to follow-up between these two waves, 670 participants successfully followed up in 2006. Finally, after excluding 42 participants with incomplete data from the 2006 wave, data from these 628 individuals were finally analyzed in the present study (Fig. 1).

### Measures

#### Cognitive function

We utilized a modified version of the Short Portable Mental Status Questionnaire (SPMSQ) as an observed variable to evaluate cognitive function [19]. This 8-item scale assessed various cognitive domains, including orientation, remote memory, and attention, with the following questions: (1) Tell me your address or the location of the site where you are being, (2) What is today’s date (including the year, month, and day)?, (3) What day of week is it?, (4) How old are you this year?, (5) What is your mother’s maiden name?, (6) Who is the current president?, (7) Who was the president before him? The final item includes a serial 3 s subtraction assessment where the respondent starts with 20 and subtracts by 3 twice iteratively. Each response is scored using a binary system, where a correct answer receives a score of 1, and an incorrect response receives a score of 0. The total scores are then calculated to reflect the cognitive levels of the respondents, ranging from 0 to 8. A lower total score indicated a more severe degree of cognitive impairment. The internal consistency of the SPMSQ, as measured by Cronbach’s alpha was 0.75.

#### Disability (Mobility, IADL, ADL)

Disability was utilized as a latent variable, comprising three observed variables: mobility, Instrumental Activities of Daily Living (IADL), and Activities of Daily Living (ADL).

##### Mobility

The mobility assessment is a 4-point scale ranging from 0 to 3 (0 = No difficulty, 1 = Some difficulty, 2 = Great difficulty, 3 = Unable to do it). Participants were asked the following questions: “*If no one helps you*,* and you do not use aids*,* would you have difficulty doing the following activities by yourself?*”. A total mobility score was calculated by adding up scores from nine items including standing continuously for 15 min, standing continuously for 2 h, squatting, raising both hands overhead, grasping/turning objects with fingers, lifting 11–12 kg, running a short distance, walking 200–300 m, and climbing 2–3 flights of stairs. While difficulties caused by temporary health problems, or diseases with rapid recovery are not considered into having difficulties. For squatting, raising both hands overhead, and grasping/turning objects with fingers, respondents who couldn’t determine the level of difficulty were encouraged to conduct the activities on site. The total scores were then calculated, which ranged from 0 to 27. A higher total mobility score indicated more limitations in mobility within daily life. The internal consistency of these mobility items, measured using Cronbach’s alpha, was 0.87.

##### Instrumental activities of daily living (IADL)

Instrumental Activities of Daily Living (IADL) was assessed through self-reports of difficulty or the need for assistance with instrumental daily activities [20]. Respondents were asked if they had trouble doing specific daily activities such as buying personal items, managing finances/paying bills, using public transportation, doing physical household work, performing light tasks at home, and making phone calls. They could choose from multiple options, including 0 = No difficulty, 1 = Some difficulty, 2 = Great difficulty, and 3 = Unable to do it. Difficulties derived from specific conditions like illiteracy or lack of knowledge were not considered as having difficulties. A higher total IADL score indicated a greater level of instrumental limitations in daily life. The internal consistency of IADL items, assessed using Cronbach’s alpha, was 0.80.

##### Activities of daily living (ADL)

Activities of Daily Living (ADL) was measured through self-reported assessments of difficulty or the need for assistance with fundamental daily tasks [21, 22]. Respondents were asked if they had trouble doing specific daily activities, such as bathing, dressing, eating, getting out of bed/standing, moving around the house, and going to the toilet. They could choose from multiple options, including 0 = No difficulty, 1 = Some difficulty, 2 = Great difficulty, and 3 = Unable to do it. While difficulties caused by temporary health problems, or diseases with rapid recovery are not considered into having difficulties. In cases where respondents couldn’t determine the level of difficulty for eating, getting out of bed/standing, or moving around the house, they were encouraged to try these activities on the site. A higher total ADL score indicated a greater degree of functional limitations in daily life. The internal consistency of these ADL items, as measured by Cronbach’s alpha, was found to be 0.81.

### Statistical analysis

Descriptive statistics, including means and standard deviations for continuous variables, and frequencies with percentages for categorical variables, were calculated. To assess differences in baseline characteristics, cognition, and disability, chi-square tests and t-tests were employed for categorical variables and continuous variables, respectively. Associations between variables were examined through Pearson correlation adjusted for demographic factors.

To comprehensively examine the relationship between cognition and disability, we employed structural equation modeling (SEM) and cross-lagged panel analysis. One observed variable representing cognitive function (SPMSQ) and a latent variable representing disability, which consists of three observed variables (mobility, ADL, and IADL) were analyzed within the SEM framework. Cross-lagged panel analysis was used to examine whether baseline disability or cognitive impairment was associated with each other between 2000 and 2006. The full information maximum likelihood (FIML) method was used in our analysis to take missing values into consideration [23].

**Fig. 2:**
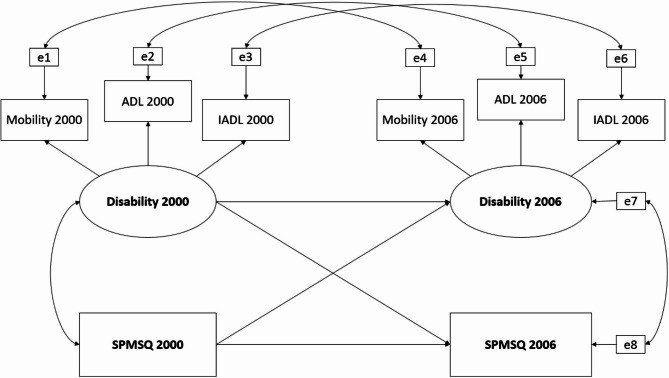
Conceptual model of cross-lagged relationship between cognitive function and physical function

The construction of the cross-lagged model was built stepwise as follows. First, we built measurement models for disability, comprising three measures: mobility, ADL, and IADL in both the 2000 and 2006 waves through confirmatory factor analysis (CFA). Additionally, we set up error covariance for the same observed variables across the two waves due to inherent data autocorrelation. Secondly, we constrained the factor loadings of the same observed variables across two waves to ensure the consistency of the underlying construct. Finally, we constructed the two-wave cross-lagged model. The conceptual model is presented in Fig. 2.

The p-value of a path less than 0.05 indicates a significant association, and standardized coefficients were applied to quantify the extent of correlations. To assess the model fit, various fit indices were utilized, including the χ2 test, comparative fit index (CFI), root mean square error of approximation (RMSEA), and standardized root mean square residual (SRMR). An acceptable fit was indicated by using CFI ≥ 0.95, RMSEA < 0.1, and SRMR ≤ 0.08 [24, 25]. The data were analyzed by the SAS 9.4. statistical software [26].

## Results

### Demographic data of the sample data

The demographic characteristics of the sample data are summarized in Table 1. Of the 628 participants, 373 participants (59.39%) were male, and 255 participants (40.61%) were female. The average age of the participants in 2000 was 66.0 years (SD = 7.31). The mean scores for IADL, ADL, and mobility in 2000 were 0.65(SD = 1.79), 0.06(SD = 0.57), and 2.80(SD = 4.32), respectively, providing insight into baseline physical function. In the second wave in 2006, the mean scores5. for IADL, ADL, and mobility were 2.02(SD = 3.75), 0.55(SD = 2.31), and 5.13(SD = 6.31), respectively. Additionally, the mean SPMSQ score in 2000 was 7.81(SD = 0.39), indicating a generally high level of cognitive function among participants. By 2006, the mean SPMSQ score in 2006 was 7.39(SD = 1.10). Both disability (ADL, IADL, mobility) and cognition (SPMSQ) showed a decline over time.

**Table 1:** Participant characteristics at baseline and follow-up (SEBAS 2000 and SEBAS 2006)

Variable	SEBAS2000	SEBAS2006	Paired t or χ2	P value
Age	66.0±7.31	71.93±7.20	-235.01	<0.0001
Sex(Male/Female)	373/255	373/255		
Education(n/%)				
No schooling	159(25.32)	159(25.32)		
Elementary(<6yrs)	279(44.43)	279(44.43)		
Junior High(7-9yrs)	71(11.31)	71(11.31)		
Senior High(9-12yrs)	68(10.83)	68(10.83)		
College(>12yrs)	51(8.12)	51(8.12)		
Disability				
IADLs	0.65±1.79	2.02±3.75	-10.82	<0.0001
ADLs	0.06±0.57	0.55±2.31	-5.72	<0.0001
Mobility	2.80±4.32	5.13±6.31	-11.49	<0.0001
Cognitive impairment				
SPMSQ	7.81±0.39	7.39±1.10	9.75	<0.0001

*IADLs* Instrumental Activities of Daily Living, *ADL* Activities of Daily Living, *SPMSQ *Short Portable Mental State Questionnaire

### Confirmatory factor analysis and cross-lagged panel analysis

**Fig. 3:**
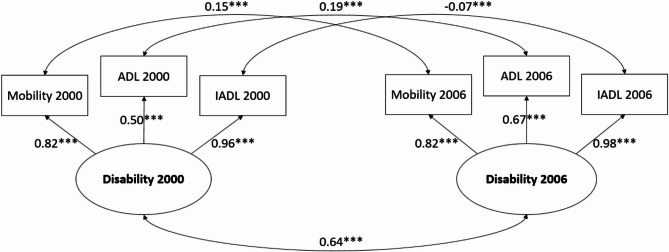
Confirmatory factor analysis without constraints on factor loadings of latent variables. ***p < 0.001

Our proposed measurement model was constructed with a latent variable, disability, composed of ADL, IADL, and mobility. We also introduced error covariance between the same variables across two waves, taking data autocorrelation into account. The CFA model demonstrated a strong model fit, with all the standardized factor loadings of all observed variables being statistically significant (χ2 (3) = 10.16, *p* = 0.02, RMSEA = 0.06, CFI = 1.00, SRMR = 0.02). Subsequently, we imposed constraints on the factor loadings of latent variables, making them equal across two waves. However, the model fit indices revealed a suboptimal fit under these constraints (χ2 (5) = 141.68, *p* < 0.001, RMSEA = 0.21, CFI = 0.94, SRMR = 0.13). Therefore, the CFA model without constraints was used for the subsequent cross-lagged analysis. The detailed results from the final CFA model are presented in Fig. 3.

**Fig. 4:**
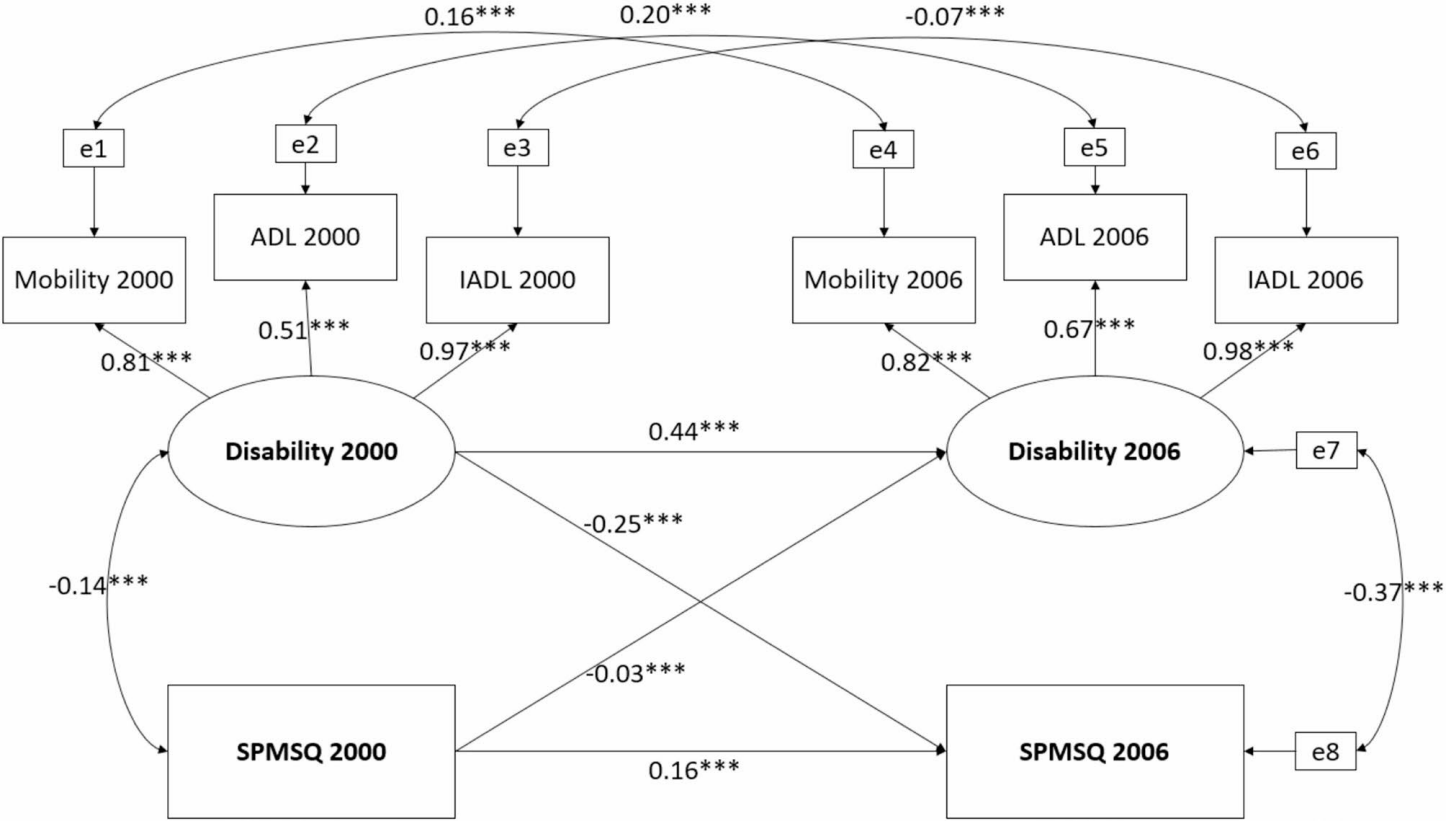
Associations between physical function and cognitive impairment with cross-lagged analysis. ***p < 0.001

The cross-lagged model was constructed with a good fit(χ2 [9] = 34.59, *p* < 0.001, RMSEA = 0.0673, CFI = 0.99, SRMR = 0.03). In 2000, a negative association was observed between cognitive function (SPMSQ) and disability (*r*=−0.14, *p* = 0.004) (Fig. 4). The standardized coefficient of autoregressive paths of cognitive impairment was 0.16 (*p* < 0.001), while for disability over the same period, the coefficient was 0.44 (*p* < 0.001). Both cross-lagged paths were statistically significant (*p* < 0.001), with standardized coefficients of −0.03 for cognitive impairment to future disability and − 0.25 for disability to future cognitive impairment. Additionally, Fisher’s Z test was used to compare the two cross-lagged coefficients, and the result was statistically significant (*p* < 0.001).

## Discussion

This study revealed a bidirectional relationship between cognitive change and disability. The findings indicate that baseline cognitive function was positively associated with cognition function, but negatively associated with disability six years later. Similarly, baseline disability was positively associated with disability, but negatively associated with cognitive function 6 years later, indicating that those with disabilities at baseline did have a worse cognitive function at follow-up. Furthermore, the Fisher Z test confirmed that the path from disability to future cognitive impairment was stronger than the path from cognitive impairment to future disability.

Inconsistent measurements of disability and cognition may have led to different results in previous studies on the relationship between these two conditions. For disability, Sun et al. categorized ADL domains, such as bathing and feeding, into a hierarchy representing early, intermediate, and late ADL loss to capture ADL dysfunction [17]. In contrast, Krall et al. assessed physical function using measures like gait speed, chair stands, and balance, focusing primarily on lower extremity strength [14]. In the present study, we considered disability as a concept that exists on a continuum and encompasses various tasks in diverse daily settings, rather than physical function alone [27, 28]. Therefore, we incorporate multiple facets of disability-ADL, IADL, and mobility into a latent variable, to better represent the complexity of disability, which may explain the differences in our findings compared to previous studies. Similarly, differences in how cognition is measured could also account for inconsistency across studies. While some studies use global cognitive measures like the Mini-Mental State Examination(MMSE) or SPMSQ, others use more domain-specific tests(memory, executive function).

In the present study, the factor loadings of ADL in 2000 and 2006 from disability were 0.50 and 0.67, respectively, which are lower than those of IADL and mobility. While factor loadings of 0.7 or higher are generally preferred, values between 0.50 and 0.70 are still considered acceptable in social science research [29, 30]. A similar situation was also observed in Sun’s study, where factor loadings from the hierarchy of ADL loss to each domain ranged from 0.46 to 0.96 [17]. This suggests lower factor loadings may be common when measuring disability, a construct that is inherently complex and multifaceted. Additionally, Field et al. point out that even lower factor loadings can be acceptable if the overall model fit is strong, as shown in our study [31]. The moderate factor loadings of ADL may reflect its limited ability to capture the full scope of disability, further reinforcing the idea that disability is a multidimensional concept, which cannot be sufficiently represented by a single measure like ADL alone.

Age and life stages can significantly influence the relationship between disability and cognitive change. A longitudinal study from the Netherlands found that cognitive function before the age of 75 was not a reliable predictor of future physical function [15]. However, after age 85, poorer cognitive performance was associated with declines in gait speed and grip strength. Additionally, in comparison to individuals aged 65–74, slower gait speed in those aged 75–84 predicted a steeper decline in cognitive function. Another study using mixed regression effect models also suggested that ADL and IADL disabilities were related to accelerated cognitive impairment [32]. These findings imply that the interaction between disability and cognitive impairment may differ across life stages. In our study, given that participants in both SEBAS 2000 and 2006 cohorts maintained relatively preserved physical function, and that we excluded those with cognitive impairment at baseline, our result emphasizes the interaction of disability and cognitive change during the early stages of both conditions’ progression [33]. This may explain why our analysis revealed a predominant cross-lagged path from disability to future cognitive impairment, while the previous studies didn’t. In addition to these reasons, factors such as participant characteristics, follow-up duration, and variation in methodology could also contribute to differences across studies.

Lifestyle factors may partially explain the bidirectional relationship between disability and cognitive impairment. Individuals with disability or cognitive impairment often face challenges in engaging in physical activities, whether due to physical limitations or a lack of willingness [34]. Physical activities, however, have been shown to slow the progression of disability and cognitive impairment [35, 36]. One study even identified physical activity as a significant mediator between cognitive change and physical function [13]. Additionally, social participation and mentally stimulating activities, such as household chores, playing chess, and interpersonal interactions, are essential in reducing cognitive decline. Physical limitations related to disability can lead to increased social isolation and reduced access to cognitive activities, both of which contribute to cognitive decline [37–43]. This broader impact of disability on social engagement and mental stimulation may help explain why, in our study, baseline disability had a stronger effect on future cognitive impairment than baseline cognitive function had on future disability. Furthermore, people with disability or cognitive impairment may lose some self-care capacity, leading to unhealthy lifestyle habits, which exacerbate the risks for both conditions [44]. Healthy dietary patterns featuring fruits, vegetables, and protein benefit both physical function and cognition [45–48]. Previous research also suggests a potential link between malnutrition and cognitive impairment, as people with mild cognitive impairment have been found to have lower levels of whole-blood magnesium [49]. A large-scale randomized controlled trial involving 1260 older adults assigned to either an intensive multidomain intervention group or a control group examined the effects of dietary modifications, regular exercise, and cognitive training. The results showed that the intervention group had significantly greater improvement in executive functioning and global cognition after a two-year follow-up, providing high-level evidence that lifestyle changes play a critical role in slowing the progression of disability and cognitive impairment [50]. Future research should further explore how lifestyle factors mediate the relationship between cognitive function and disability to better understand the bidirectional nature of this association.

The lifestyle habits of older adults in Taiwan may partly explain the association observed among our participants in this study. A significant proportion, 42.7%, of older adults in Taiwan do not regularly exercise over three times a week, and 37.1% never exercise at all. Among those who exercise regularly, around 34% spend less than 30 min per session. Additionally, the most favorable exercise was strolling, which is a low-intensity activity with limited effects on improving physical function [51]. This indicates that many older adults in Taiwan fall short of the exercise recommendations set by the WHO [52, 53]. Furthermore, the average sedentary time among older adults in Taiwan was 609.74 min per day, which is obviously higher than in other countries (e.g. Japan: 462 min per day) [54–57]. Prolonged sedentary behavior can lead to significant declines in physical function [54, 58]. Despite this, older adults in Taiwan spend an average of 2 h and 45 min watching TV and 20 min reading newspapers or magazines [59]. Interestingly, these sedentary behaviors, such as reading or watching TV, may have a protective effect against cognitive decline [60]. These findings suggest that due to cultural and lifestyle factors, older adults in Taiwan may experience a greater decline in physical function compared to cognitive function, a trend that was also observed among our study participants. This could partially explain why, in our cross-lagged analysis, the pathway from disability to future cognitive impairment was more prominent than the reverse pathway from cognitive impairment to future disability.

This study has several strengths. First, it employed a longitudinal design with a nationally representative cohort, providing evidence of bidirectional relationships between disability and cognitive impairment. The findings also highlight that disability plays a more significant role in the aging process than cognition, suggesting that routine screening for disability status in older adults may be more effective in preventing cognitive decline. Moreover, this study offers a more comprehensive approach to understanding of disability by utilizing a latent variable to capture the full spectrum of disability change, surpassing previous research that focused solely on physical performance.

This study has several limitations. First, it analyzed only 2 waves of data from 2000 to 2006, which may limit the findings to a specific life stage and restrict their ability to capture long-term trends between cognitive impairment and disability. Second, only 74.8% of participants completed the second-wave interviews and examinations in 2006, raising the possibility that the results may reflect a healthier subset of the original sample. Third, relying on the SEBAS database, which includes only Taiwanese participants, may limit the generalizability of the findings to other populations. Fourth, the modified version of the SPMSQ used in this study assessed only certain cognitive domains rather than global cognitions. Finally, many variables were based on self-reported survey data, potentially leading to response bias. Future studies could reduce this bias by using objective measures, such as conducting on-site assessments of disability under clinician observation.

## Conclusions

This longitudinal study underscores the bidirectional relationship between disability and cognitive impairment in older adults, with disability exerting a stronger influence on future cognitive impairment has on future disability. These findings suggest that interventions focused on preventing and managing disability in older adults could help reduce the risk of subsequent cognitive impairment. Additionally, the study further explores how lifestyle factors, including physical inactivity and sedentary behaviors, contribute to this relationship in older populations in Taiwan.

## Data Availability

Data from SEBAS study was publicly accessible with a request to the Ministry of Health and Welfare, Taiwan.

## Abbreviations

ADL: Activities of Daily Living
IADL: Instrumental Activities of Daily Living
SPMSQ: Short Portable Mental Status Questionnaire
SEBAS: Social Environmental and Biomarkers of Aging Study
CFA: Confirmatory Factor Analysis
RMSEA: Root Mean Square Error of Approximation
CFI: Comparative Fit Index
SRMR: Standardized Root Mean Square Residual
FIML: Full Information Maximum Likelihood
WHO: World Health Organization
